# Radiation-induced morphea of the breast: a case report

**DOI:** 10.1186/1752-1947-2-136

**Published:** 2008-04-30

**Authors:** Nellie LC Cheah, Daniel WY Wong, Anula D Chetiyawardana

**Affiliations:** 1 Cancer Centre, Queen Elizabeth Hospital, University Hospital Birmingham NHS Trust, Edgbaston, Birmingham B15 2TH, UK

## Abstract

Radiation-induced morphea (RIM) of the breast is a rare complication of radiotherapy. It is disfiguring, painful and defeats the purpose of achieving a good cosmesis in breast-conservation surgery. This report describes a severe case of RIM in a breast cancer patient together with photographic illustrations of the serial changes over time and histopathology slides. A review of the literature is provided.

## Introduction

Breast cancer is the most common cancer in women with an incidence of 134 per 100,000 women in the United Kingdom, and the incidence is still rising [1]. Radiotherapy to the breast following breast-conservation surgery reduces local recurrence and improves survival [2]. Radiotherapy can cause acute skin reactions such as erythema or moist desquamation, especially in women with large breasts. Late side effects may include fibrosis (10%), skin telangiectasia (10%), atrophy (8%) and pain (2%) [3]. We present a case of radiation-induced morphea (RIM) of the breast, a rare late side effect of radiotherapy, with serial changes in the appearance over a period of 3 and a half years.

## Case presentation

In October 2000, a 57-year-old Caucasian woman was diagnosed with breast carcinoma following mammogram screening. She had a wide local excision of the left breast and axillary node sampling. Histology was reported as T1N0 grade 1 invasive ductal carcinoma with clear resection margins. She was treated with tamoxifen 20 mg daily and commenced on adjuvant radiotherapy to the left breast and axilla. The radiotherapy dose was 40 Gy in 15 fractions using medial and lateral tangential fields of 5 MV photons followed by 5 Gy in two fractions of 10 MeV electron boost to the tumour bed.

**Figure 1:**
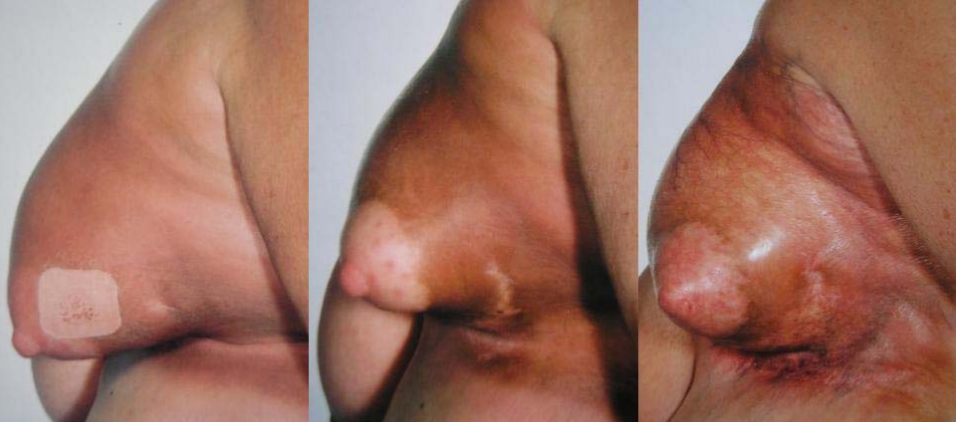
**Changes in the left breast over time**. Nine months post-radiotherapy, the breast was erythematous, swollen and looked bruised (left panel). One year post-radiotherapy, the breast was retracted and pigmented (middle panel). Three and a half years post-radiotherapy, the breast had reduced in size and hardened with hypo- and hyper-pigmentation. The inframammary fold had significant skin telangiectasia (right panel).

At the end of her radiotherapy treatment the patient developed acute radiotherapy reaction in the left inframammary fold consisting of mild moist desquamation that subsequently resolved. On clinic review nine months later, she noticed a spontaneous, painful and discoloured swelling of the left breast (Figure 1, left panel). This was initially diagnosed as cellulitis and treated with antibiotics, but as there was no improvement, a fine-needle aspiration was carried out which showed no recurrence of the breast cancer.

She was referred to a rheumatologist and dermatologist. She was screened for systemic collagen disease and fungal infection. By then, the abnormal area had extended further to the axilla and medial aspect of the left upper arm. A review of the breast radiotherapy plans and delivery did not reveal any incorrect delivery or unexpected high subcutaneous dose.

**Figure 2:**
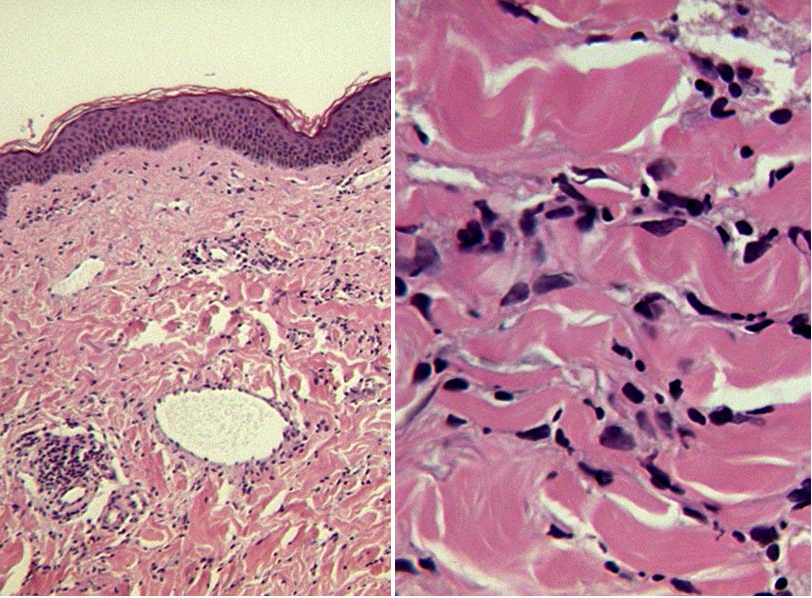
Histopathology slides showing lymphocytic infiltrate and thickened collagen bundles in the dermis.

Subsequent breast biopsies confirmed RIM of the breast. The histology showed intact epidermis but the papillary dermis had a patchy perivascular lymphocytic infiltrate. The most striking changes were in the reticular dermis where the collagen bundles appeared thickened with increased eosinophilia, accompanied by a moderate amount of chronic inflammatory cell infiltrate consisting of lymphocytes predominantly, plasma cells and histiocytes (Figure 2).

Various treatments including topical and oral steroids and oral psoralen ultraviolet-A (PUVA) therapy did not improve the appearance of the breast. Paracetamol and amitriptyline were used to control her breast pain. The patient was referred to a plastic surgeon but she declined any reconstructive surgery.

The photographs in Figure 1 show the changes in the left breast over time. The initial appearance was of swelling, erythema and bruising (Figure 1, left panel). It reduced in size and became harder with skin telangiectasia over one year (Figure 1, middle panel) and 3 and a half years post-radiotherapy (Figure 1, right panel). On her clinical review 6 and a half years post-radiotherapy, she remained free from recurrence and the breast appearance did not deteriorate any further.

## Discussion

RIM of the breast is rare, with an estimated incidence of 0.20% [4]. Its onset ranges from 1 to 12 months [4,5], but could be as late as 32 years post-radiotherapy [6]. Synonyms include radiation port morphea, radiation-induced scleroderma, radiation port scleroderma, post-irradiation morphea, localised scleroderma and circumscribed scleroderma.

RIM can extend beyond the irradiated area. A patient with breast cancer, with the confounding factors of endometrial cancer and pelvic radiotherapy 3 years prior, developed RIM in the breast, abdominal wall and leg at 2, 12 and 54 months respectively, following breast radiotherapy [7].

RIM is not an exaggerated post-irradiation fibrosis of the breast. Post-irradiation fibrosis is more common and is primarily a deep subcutaneous and fascial fibrosis with little or no inflammatory infiltrate [6]. In contrast, RIM is a localised scleroderma of primarily dermal fibrosis (Figure 2) due to radiation-induced disturbance in the immune system, vasculature and collagen metabolism. RIM has an initial inflammatory phase and a subsequent 'burnt-out' phase, where the latter displays induration, fibrotic retraction and pigmentation of the breast. The pathophysiology of RIM is thought to be radiation-induced neoantigen formation that subsequently stimulates secretion of transforming growth factor beta (TGF-β). TGF-β strongly induces fibroblast activation, collagen synthesis and, hence, excessive fibrosis [4,6].

There is no definite correlation between tamoxifen use and the development of RIM. However, tamoxifen can induce TGF-β secretion and increase lung fibrosis in breast cancer patients receiving both tamoxifen and radiotherapy [8].

Systemic sclerosis is a relative risk factor for developing an exaggerated post-irradiation fibrosis, although this patient had no previous history of systemic sclerosis. Age and radiotherapy parameters such as total radiation dose, dose per fraction and severity of acute reaction do not seem to be significant risk factors for developing RIM [5].

Various treatments including oral antibiotics and topical, intralesional or systemic steroids have been used in treating RIM of the breast with varying results [5]. One patient had improvement without any treatment [6].

Morphea has been treated with topical and systemic steroids, colchicine, D-penicillamine, immunosuppressants and cytotoxics such as azathioprine, methotrexate, cyclophosphamide and cyclosporin, plasmapheresis and extracorporeal photopheresis, with variable benefit [9].

PUVA therapy in 10 to 20 sessions for morphea has been reported to cause remarkable skin softening associated with a reduction in skin cytokines including TGF-β [9]. The mechanism of action of PUVA is attributed to the suppression of inflammation and collagen mediators causing a reduction in pruritus, pigmentation and skin tightness.

Any treatment modality should be started promptly to give the best outcome, as fibrosis and atrophy are not reversed by PUVA therapy. Early referral to a dermatologist is therefore recommended.

Reconstructive surgery for morphea of the breast has been reported to give a good cosmetic result [10]. To the best of the authors' knowledge, no surgery has yet been performed for RIM of the breast.

## Conclusion

Adjuvant radiotherapy following breast-conservation surgery can reduce local recurrence and improve survival in breast cancer patients. A late radiotherapy side effect in the form of RIM of the breast is rare, but causes significant morbidity and deformity. Clinicians need to be aware of RIM after ruling out infection or recurrence of breast cancer, as RIM can mimic these conditions. There is no proven treatment once the condition is established. There are no reliable predictors for the development of RIM, and its occurrence defeats the purpose of achieving good cosmesis in breast-conservation surgery.

## Competing interests

The authors declare that they have no competing interests.

## Authors' contributions

NLCC was primarily responsible for the drafting, submission and revision of the manuscript and the literature search. DWYW and ADC were responsible for editing the manuscript and provided advice during the literature review. All authors have read and approved the final manuscript.

## Consent

Written informed consent was obtained from the patient for publication of this case report and accompanying images. A copy of the written consent is available for review by the Editor-in-Chief of this journal.
